# Idealized analysis of relative values of bidirectional versus unidirectional electric vehicle charging in deeply decarbonized electricity systems

**DOI:** 10.1016/j.isci.2022.104906

**Published:** 2022-08-11

**Authors:** Michael O. Dioha, Tyler H. Ruggles, Sara Ashfaq, Ken Caldeira

**Affiliations:** 1 Department of Global Ecology, Carnegie Institution for Science, Stanford, CA 94305, USA; 2 Breakthrough Energy, Kirkland, WA 98033, USA

**Keywords:** Energy resources, Energy policy, Energy systems, Energy management, Energy modeling

## Abstract

We employed an idealized macro-energy system model to examine how the value of unidirectionally- and bidirectionally-charging electric vehicles (EVs) varies with EV penetration and mix of electricity generators. We find that EVs can help wind and solar-based electricity generation systems to be less costly by making better use of power that would otherwise be curtailed and, potentially, by giving electricity back to the grid at times of peak net load. At low levels of EV penetration, bidirectional EVs are valuable because they can provide electricity at times of main load peak. At today’s low levels of EV penetration, bidirectional EVs stimulate investments in solar and wind generation and substantially reduce the need for grid-battery storage compared to unidirectional EVs. At high levels of EV penetration, generation capacity must be increased, and most peaks in main net load demand can be met by reductions in charging by unidirectional EVs.

## Highlights

• Electric vehicles (EVs) reduce average generation costs for solar and wind systems
• Unidirectional flexibly charging EVs can use underutilized generating capacity
• Bidirectionally charging EVs can decrease need for added solar and wind generation
• The marginal value of bidirectional EVs decreases with added unidirectional EVs

## Graphical abstract

**Figure:**
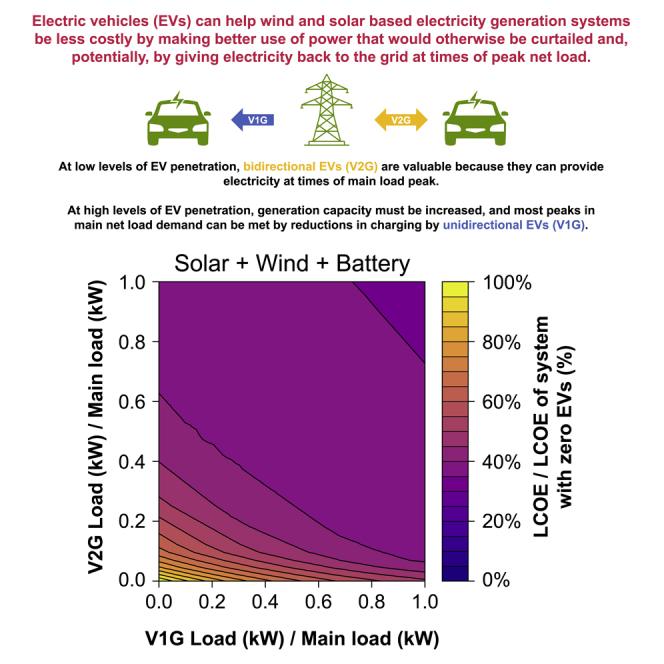

## Teaser

Energy resources; Energy policy; Energy systems; Energy management; Energy Modelling

## Introduction

Electrification, driven by decreasing costs and increasing incentives to limit climate change, may profoundly transform the transportation energy system in the coming years. Consequently, there is now considerable interest in the adoption of electric vehicles (EVs) in different regions of the world with the goal of mitigating transportation-related emissions (Dioha et al., 2022a, 2022b; Tamor and Stechel, 2022). The number of EVs on the world’s roads has increased from close to zero in 2010 to over 10 million in 2020, with battery electric vehicles leading the expansion (IEA, 2022a). In its 2021 Global EV Outlook, the International Energy Agency (IEA) projects the number of EVs to reach 145 million by 2030—accounting for around 7% of the total road vehicle fleet in that year (IEA, 2022b). This increasing EV deployment, plus the increasing needs of a growing and economically developing global population, will likely increase global electricity demand. To achieve net-zero carbon emission goals while electricity demands increase, the power sector would need to completely decarbonize, or offset residual emission with now-costly atmospheric carbon dioxide removal. While some analyses that consider current costs see a role for nuclear power in least-cost carbon-emission-free electricity systems (Duan et al., 2022), solar and wind energy will lead the power sector decarbonization agenda, according to the International Renewable Energy Agency (IRENA, 2018).

Solar and wind resources are characterized by the variability of supply on daily, weekly, and annual timescales (Antonini et al., 2022). Consequently, electricity production mixes dominated by solar and wind may require electricity storage systems to balance the possible mismatches between electricity supply and demand (Gielen et al., 2019; Tambari et al., 2020). To achieve low carbon energy systems in the future will require substantial investments in clean technologies now and in the near future. Approaches to replace system flexibility—now provided primarily by natural gas—will be needed as fossil fuel CO_2_ emissions are phased out (Ruggles et al., 2021).

At present, this mismatching challenge is mainly solved by fossil-based dispatchable electricity generators (e.g., natural gas plants) (Bellocchi et al., 2019b). If future energy systems continue to decarbonize via solar and wind, and carbon dioxide (CO_2_) emissions become increasingly constrained, there may not be enough fossil-based dispatchable generation available to fill the gap during periods of low solar and wind electricity generation and/or high electricity demand (Tong et al., 2020, 2021). Battery storage has been seen as an option to support non-dispatchable solar and wind, but the cost-effectiveness of utility-scale battery energy storage remains, arguably, a difficult hurdle to cross (Comello and Reichelstein, 2019; Tong et al., 2020). However, the batteries in EVs have the potential to serve as distributed storage systems that can be employed for multiple purposes (Englberger et al., 2021). Automobiles are used for transportation on average only 5% of the time (Fengler, 2020). In this context, the growing number of EVs can doubly support the global energy transition. They can reduce the demand for fossil fuels in the transportation sector, and they can also provide a storage option for electricity systems dominated by variable renewable energy sources (VRES) (Kühnbach et al., 2021).

In general, EV charging can occur in two ways: (i) 1-way, unidirectional charging, and (ii) 2-way, bidirectional charging (Bampanga et al., 2020). With appropriate charging strategies, EVs can effectively support a transition to cost-effective low-emission energy systems (Dioha et al., 2022a, 2022b). When EVs are charged per the drivers’ charging habits without regard to the electricity system’s needs, the result may be an increase in peak power demand and increased curtailment of VRES generation. However, when the drivers’ charging strategy aligns with the power network needs, EVs can become a potential asset for the grid. EVs can support the electricity system when they are charged during hours of critical excess electricity generation from VRES, and when they supply electricity back to the power system during hours of peak electricity demand through vehicle-to-grid technology (Kühnbach et al., 2021).

Many studies have investigated the impact of EV charging strategy on the electricity system. The value of EV in terms of power system operation cost, levelized and marginal cost of electricity generation, power plant dispatch, and environmental emissions could substantially be affected by the EV charging strategy employed (Borlaug et al., 2020). The effect of EV on some of the above parameters has been examined for different countries (Bellocchi et al., 2019b, 2019a; Booysen et al., 2022; Broadbent et al., 2022; Dioha et al., 2022a, 2022b; Dumlao and Ishihara, 2022; Greaker et al., 2022; Hill et al., 2019; Jenn and Highleyman, 2022; Kejun et al., 2021; Mangipinto et al., 2022; Tarroja and Hittinger, 2021; Zhang et al., 2022). Although there are numerous other studies on the value of EVs, this paper focuses on the relative value of EVs based on the plausible circumstances of different regions in an idealized context. A snapshot of different EV studies and a comprehensive review of this subject matter are available in the literature (Muratori et al., 2021; Nour et al., 2020).

In electricity systems dominated by non-dispatchable generators (solar and wind), the potential value for coordinated EV charging may serve to provide system flexibility. In contrast, the value of this service would be different in electricity systems that are dominated by dispatchable generators, where flexibility is less valuable. Currently, EVs are deployed both in regions with electricity mixes that consist of VRES (e.g., Germany), and in regions where systems are dominated by dispatchable generators (e.g., Qatar). For future electricity zero-emissions grids, there could also be substantial differences across regions due to differences in VRES resource potential. For example, a country such as Nigeria, with huge solar potential and poor wind regime, solar may dominate a future zero-emissions grid (Dioha and Kumar, 2018; Oyewo et al., 2018; Tambari et al., 2020). However, for a country such as Denmark, wind may dominate a future zero-emissions grid (Danish Ministry of Climate Energy and Utilities, 2019).

These different regional circumstances suggest that the value of EVs in different electricity system configurations could vary substantially. Furthermore, the value of EVs in electricity systems could be affected by the EV market status, or vehicle availability, which is a function of vehicle ownership rate in a region. To illustrate, assuming all internal combustion engine vehicles (ICEVs) are replaced with EVs worldwide, regions with a high per capita vehicle ownership ratio (e.g., U.S. with 797 vehicles per 1000 inhabitants (Our World in Data, 2021)) will have more vehicles to support the power network needs, and higher EV load, compared to regions with a relatively low per capita vehicle ownership ratio (e.g., Nigeria with 31 vehicles per 1000 inhabitants (Our World in Data, 2021)). Thus, there is no straightforward answer for how the value of EV charging strategy could vary across different energy systems due to the substantial differences in electricity system configurations, and the stock of EVs. Consequently, a macro-outlook is needed to understand the value of EVs in electricity systems for a wide range of EV stock and electricity system configurations (Levi et al., 2019). In this context, we set out to answer the question:

How does the value of bidirectional charging EVs vary with the mix of electricity generators and degree of EV penetration, relative to the value of unidirectional EVs?

To address this question, we have used a transparent idealized macro-energy system model to investigate independently the potential impacts of using only unidirectional and only bidirectional charging EVs across five plausible electricity systems, in terms of electricity cost, electricity generation capacity, and variable renewable electricity curtailment as well as EV dispatch. The electricity system configurations considered are Natural Gas-only, Natural Gas + Solar + Wind, Solar + Battery, Wind + Battery, and Solar + Wind + Battery generation systems. We study the fraction of EV-to-main load as a way to parameterize a wide variety of possible energy systems. This is helpful because regional differences in total available vehicle stock directly influence how a given EV load should be interpreted with respect to the fraction of vehicle stock converted to EVs. Thus, if the United States reached 100% EV penetration at their current vehicle ownership rate, this would be reflected in a system with an EV-to-main load ratio of 0.24, while for Australia the ratio would be 0.18 (see STAR methods section).

Our interest in this study is to understand system characteristics and to portray dynamic relationships that exist in both existing and plausible future energy systems. Consequently, we have used current costs Table 1) for all electricity generating assets and storage technologies. We consider lossless transmission & distribution of electricity. This assumption tends to favor distributed variable generators such as solar and wind power. Our analysis is anchored in the estimation of the balance of system costs, given that some number of EV batteries is provided to the electricity supply system. The intention of this paper is to provide a macro-outlook on the value of EVs which can act as a complement to country/regional EV studies with detailed grid mixes but not a replacement for detailed country/regional analysis. We do not focus on any specific region in the modeling. Our study exists in its own domain as an idealized analysis that can have wide applicability with meaningful insights for a general understanding of the value of EVs. Additionally, for countries/regions without detailed analyses, this study could be used to provide a basic understanding of what could be expected from a detailed analysis.

**Table 1:** Techno-economic assumptions for electricity technologies

Economic parameter	Solar PV	Wind	Combined-cycle gas turbine	Utility-battery storage
Fixed capital cost $\left(\$/kW_{e}\right)$	1248 (U.S. EIA, 2021b)	1846 (U.S. EIA, 2021b)	957 (U.S. EIA, 2021b)	307 $\left(\$/kWh_{e}\right)$ (U.S. EIA, 2021b)
Fixed O&M cost $\left(\$/yr.kW_{e}\right)$	15.33(U.S. EIA, 2021b)	26.47 (U.S. EIA, 2021b)	12.26 (U.S. EIA, 2021b)	24.93 (U.S. EIA, 2021b)
Lifetime (yr)	25 (IEA, 2020)	25 (IEA, 2020)	30 (IEA, 2020)	10 (IEA, 2020)
Heat rate (Btu/kWh)	–	–	6370 (U.S. EIA, 2021b)	–
Fixed hourly cost^a^$\left(\$/h.kW_{e}\right)$	0.0139	0.0210	0.0102	0.0078 $\left(\$/h.kWh_{e}\right)$
Relative efficiency	–	–	54%	90% round-trip (Lazard, 2019)
Variable O&M cost $\left(\$/kWh_{e}\right)$	0	0	0.0019	0 (captured in fixed O&M)
Variable fuel cost^b^$\left(\$/kWh_{e}\right)$	–	–	0.0191 (U.S. EIA, 2021b)	–
Total variable cost^c^$\left(\$/kWh_{e}\right)$	0	0	0.0210	0

a Calculations are based on our assumed discount rate of 7%.

b The variable fuel cost for the combined-cycle plant was based on $3/MMBtu natural gas.

c Calculations are based on the variable O&M and variable fuel cost.

**Figure 1:**
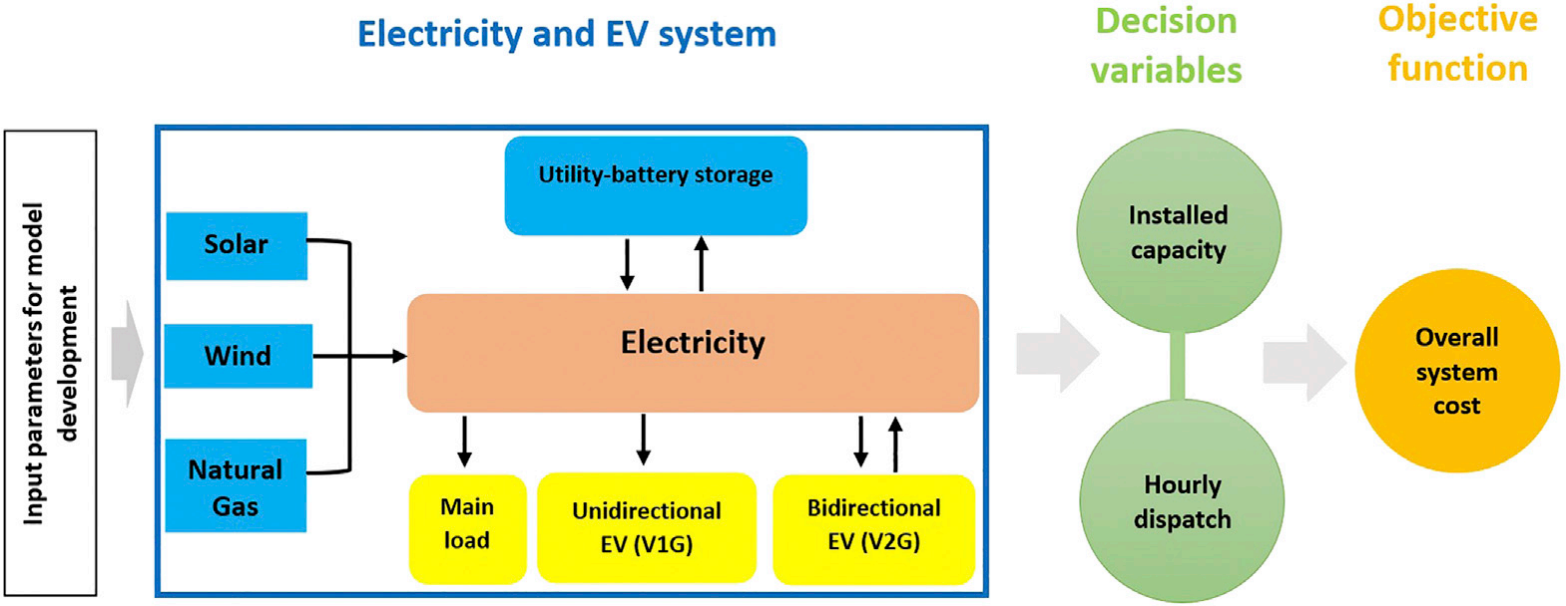
An overview of the modeling framework employed in this paper

An overview of the modeling framework applied in this paper is illustrated in Figure 1, and expatiated in the STAR methods section. This study has employed a transparent and relatively simple macro-energy system model to define various electricity generation scenarios coupled with EV (Dowling et al., 2020; Rinaldi et al., 2021; Ruggles et al., 2021; Yuan et al., 2020). Our modeling approach is idealized, and it is based on a least-cost linear optimizer that minimizes the total system cost based on the user-defined constraints. An idealized macro-energy system model has been used so we can profile a large swath of parameter space easily to understand the characteristics of the different systems studied without having to describe the complex details of EVs, power plants, and distribution lines, which could take hours (or days) to run a single simulation. The role and application of this type of macro-energy system modeling approach has been explained in detail in the study by Levi et al. (2019). For all technologies included, the model solves for the dispatch and installed capacities hourly. The unidirectional charging EV is modeled as a storage technology that draws power from the main node (grid) to satisfy only the EV load at every given hour. The bidirectional charging EV is modeled as a storage technology that draws power from the main node to satisfy the EV load and supplies power back to the main node. Analysis for the EVs was conducted separately for unidirectional and bidirectional EVs (i.e., in each simulation, either all EVs are unidirectional, or all are bidirectional).

## Results

The results of the variety of electricity system configurations described above have been examined in terms of the changes in the levelized cost of electricity (LCOE), capacity expansion, curtailment, and energy dispatched by EVs for both unidirectional and bidirectional charging EVs. To be concise, we term the unidirectional EVs as V1G, or one-way vehicle-from-grid, and the bidirectional EVs as V2G, or two-way vehicle-to-grid-to-vehicle.

### Levelized cost of electricity

**Figure 2:**
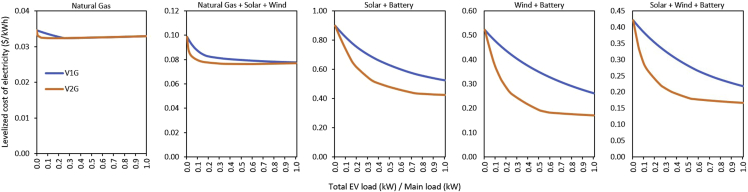
Levelized cost of electricity (LCOE): The LCOE of the system (system cost divided by the total load) is shown for unidirectional (V1G) and bidirectional (V2G) EVs. As the EV-to-main load fraction increases, the LCOE decreases across all electricity systems. This is because the flexible charging potential of EVs allows electricity systems to make better use of generation capacity, and thus reduce the system cost per total load. In the Natural Gas system, V1G and V2G achieved LCOE parity when V2G load achieved 26% of the main load. In systems consisting of solar and/or wind, there is a continuous reduction of LCOE in V2G, compared to in V1G, because V2G injects power to electricity systems that have less generating capacity, and this allows them to meet peak loads.

The levelized cost of electricity delivered (system cost divided by the total (EV + main) load) for V1G and V2G under each generation type considered is illustrated in Figure 2. For a Natural Gas- (dispatchable) generator, at modest fractions of EV penetration, an increase in EV-to-main load ($EV_{l}/M_{l}$) ratio leads to a substantial, but slower, decrease in electricity cost. In V1G, there is adequate generation capacity to cater to the EVs’ charging needs, and thus only fuel cost contributes to the electricity cost. In V2G, there is a rapid decline in electricity cost relative to V1G because the 2-way EV can help meet peak demand, reducing the need for natural gas capacity. Consequently, the electricity cost of V2G was always less than that of V1G up to when $EV_{l}/M_{l}$ reached 0.26. Beyond this point, V1G and V2G achieve electricity cost parity. That is, V2G value is no longer pronounced in the dispatchable generation system due to low variability. The only source of variability in the system is variation in load.

In the Natural Gas + Solar + Wind system (Figure 2), an increase in $EV_{l}/M_{l}$ resulted in a substantial decrease in electricity cost for both V1G and V2G. Results indicate that for the full range of $EV_{l}/M_{l}$ considered, the electricity cost of V2G remains lower than that of V1G. V2G has the capability to inject power to the grid, which reduces the need for generation overbuild and thus reduces the electricity cost compared to V1G. However, an increase in V2G penetration level reduces the effects of V2G on electricity cost because during hours of low wind and solar supply, there is still relatively less expensive natural gas capacity to provide electricity for the power network needs, as in the current US electricity systems where solar and wind variability is being smoothed out with dispatchable generators (U.S. EIA, 2021a). When $EV_{l}/M_{l}$ is 0.5 (i.e., when EV load is half of the main load), the electricity cost in V2G was just about 4% lower than the cost in V1G.

There is a relatively wide gap in the electricity cost between V1G and V2G for the Solar + Battery system (Figure 2). In V1G, electricity cost decreases across the full range of $EV_{l}/M_{l}$ options considered. In V1G, there is a relatively large amount of curtailed electricity for EV charging, which reduces the need for new capacity installation, and thus reduces the electricity cost as the stock of EV increases. In V2G, due to the 2-way flow of power, there is more incentive for cost reduction due to the additional storage provided by V2G, which offsets the cost of grid-battery storage. Consequently, V2G’s electricity cost declines further below that of V1G for the full range of $EV_{l}/M_{l}$ considered. When $EV_{l}/M_{l}$ was about 0.75, the electricity cost in V2G remains nearly the same and then gently decreases as $EV_{l}/M_{l}$ approaches 1. When $EV_{l}/M_{l}$ is 0.5, the electricity cost of V2G is about 24% lower than the cost in V1G.

The Wind + Battery system (Figure 2) shows attributes similar to those of the Solar + Battery system, but with different numerical magnitudes. Here, V1G’s electricity cost declines for the full range of $EV_{l}/M_{l}$ options considered. The electricity cost in V2G steeply declines to when $EV_{l}/M_{l}$ is about 0.6. The electricity cost of V2G when $EV_{l}/M_{l}$ is 0.5 in the Wind + Battery system was about 45% lower than the cost in V1G. The Solar + Wind + Battery system displays characteristics similar to those of the Solar + Battery and Wind + Battery systems (Figure 2). In this case, electricity cost of V2G initially starts to steeply decline to when $EV_{l}/M_{l}$ is just about 0.12. The electricity cost of V2G in Solar + Wind + Battery system is about 35% lower than the cost in V1G at 0.5$EV_{l}/M_{l}$.

To derive additional insights from our analysis, we examine scenarios where both V1G and V2G are part of the EV vehicle fleet. We seek to understand what fraction of V2G could be needed in each generator type to derive the full benefits of bidirectional EVs, in a scenario where both V1G and V2G are part of the EV stock.

**Figure 3:**
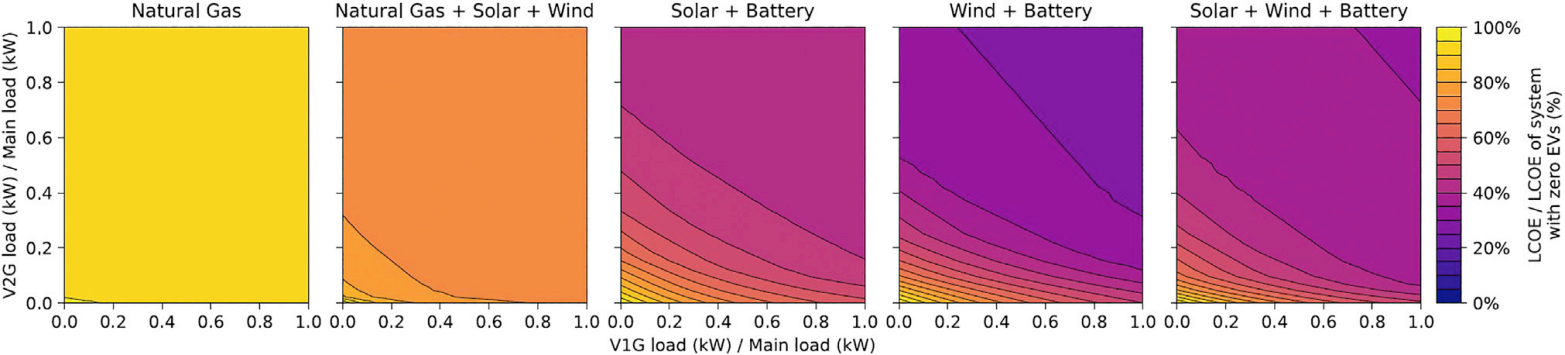
Percent reduction in levelized cost of electricity ($/kWh) from the introduction of unidirectional (V1G) and bidirectional (V2G) charging EVs in the same system: Cost reductions are greatest in systems dominated by wind and solar power. V2Gs provide substantially greater value than V1Gs, especially when total V1G penetration is low, but there is substantial benefit of having some V2Gs in the system even when V1G penetration his high. V1Gs can limit charging at times of greatest net demand, whereas V2Gs can also provide electricity to the system at times of greatest net demand.

As per Figure 3, as V2G makes up a larger percentage of the total EV stock, its value diminishes. In the Natural Gas (dispatchable generator) system, the value of increasing the percentage of V2G rapidly diminishes. It shows zero incremental value beyond 10% V2G, in our simulations at any fraction of EV-to-main-load. The four remaining systems, all powered partially or 100% by VRES, show there is value for higher fractions of V2G. However, at high EV-to-main-load fractions, diminishing returns are seen for high fractions of V2G. Our analysis suggests that in the VRES + Battery systems, the Solar + Wind + Battery system may require the least amount of V2G because a broader option of resources complements the shortfall in any resource, and this reduces the need for electricity from V2G batteries (Figure 3).

The foregoing results indicate that the proportion of V1G vs. V2G EVs plays a vital role in the value of EVs in electricity systems. Increased levels of V2G do not automatically translate to increased value for the electricity system. There is a saturation point because the level of V2G stock needed to balance power network needs varies substantially due to differences in the technology-resource configuration of different electricity systems. In sum, our results indicate that in dispatchable generator-dominated electricity systems, a small fraction of EVs with 2-way charging will attain essentially the same cost benefit as will a full V2G fleet. In zero-emission electricity systems dominated by VRES, a relatively modest number of 2-way EVs will attain maximum cost benefits, because only a modest amount of V2G battery storage is needed to mitigate solar’s day-night cycle and wind’s synoptic-scale weather variability.

**Figure 4:**
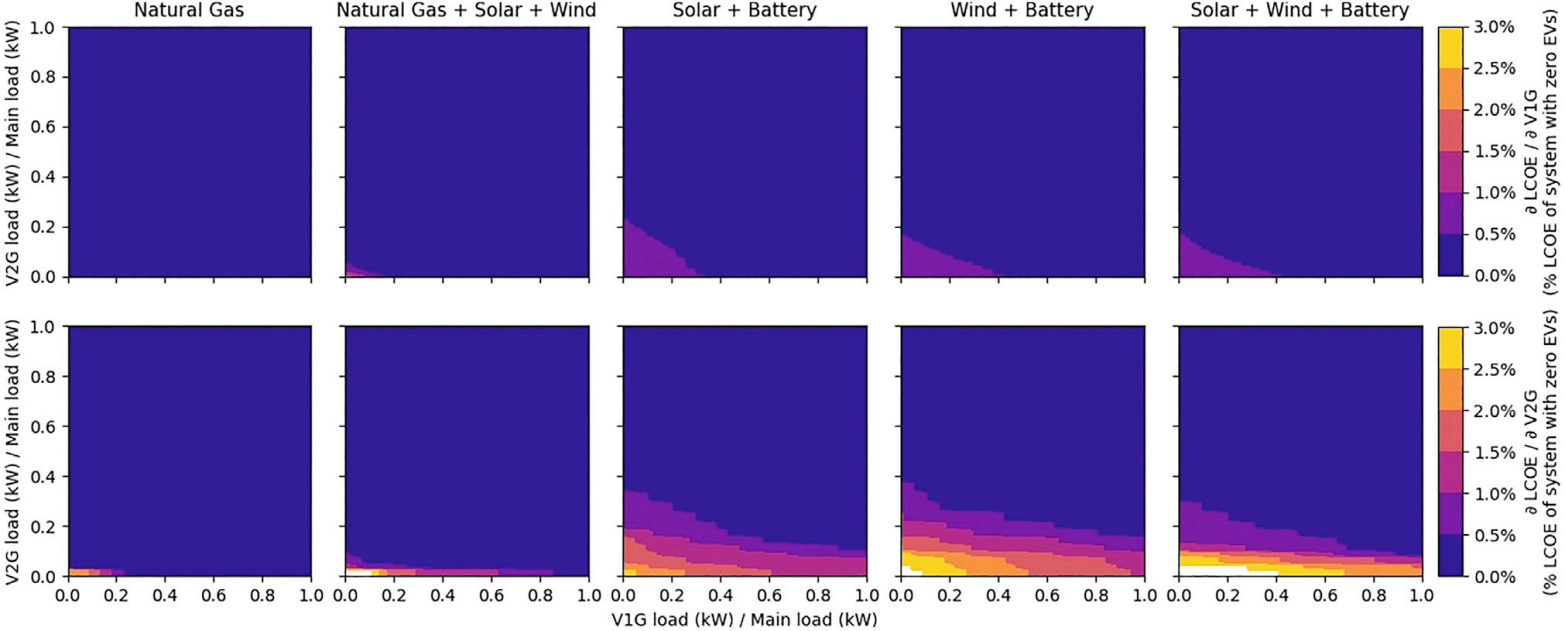
Marginal value of adding additional V1G’s (top row) or V2G’s (bottom row) at different levels of V1G and V2G penetration Values are represented as % reduction in system cost without EVs per % increase in V1G/main-load demand or V2G/main-load demand (top and bottom rows, respectively). Note the high value associated with V2Gs in systems with solar and/or wind up to the point where V2G demand is equal to 10% or 20% of main load demand. Maximum values appear at the origin in all cases, left-to-right in the top row, maximum values are 0.38%, 1.59%, 0.98%, 0.98%, and 0.98% cost reduction relative to no EVs per % increase in V1G/main-load demand; left-to-right in the bottom row, maximum values are 2.47%, 6.47%, 2.67%, 3.36%, and 4.50% cost reduction relative to no EVs per % increase in V2G/main-load demand.

The marginal value of adding more V2G in the system compared to V1G in the system is shown in Figure 4. V2Gs lower costs of delivered electricity by reducing the amount of generation capacity needed to meet peak residual (or net) loads. However, when mean V1G demand is high, additional generation capacity is needed to meet V1G demand. With increased generating capacity, and flexible charging of V1Gs, peak residual (or net) loads are smaller, and thus added V2Gs have lower value than they have at low penetration of V1Gs.

### Capacity and curtailment in different systems

**Figure 5:**
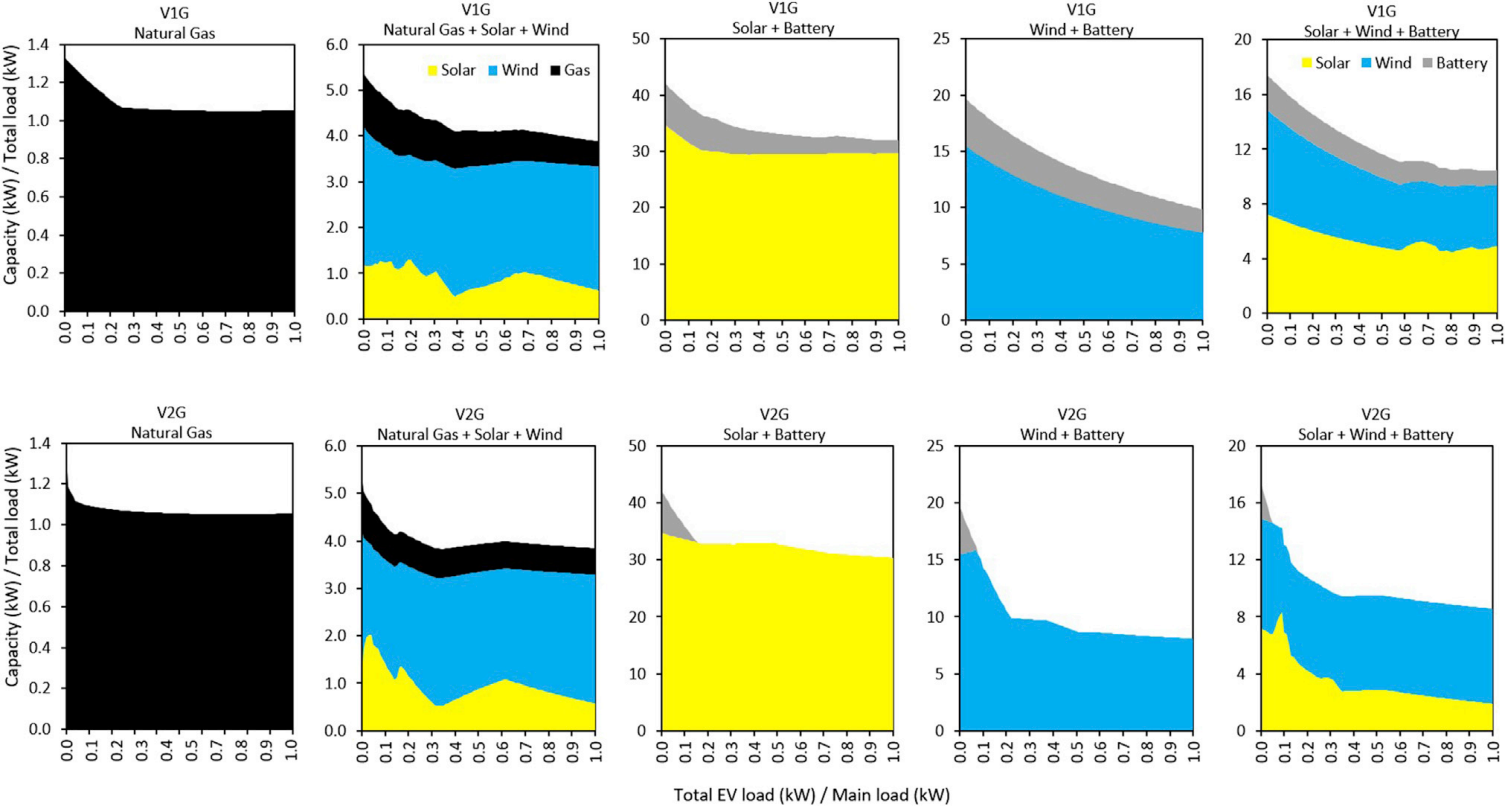
Electricity system generation capacities: the total available generation capacity divided by the total (EV + main) load is shown for unidirectional (V1G) and bidirectional (V2G) EV for the full range of EV-to-main load fraction ($EV_{l}/M_{l}$). Grid-battery capacity is delineated in kW by dividing the kWh capacity by the charging/discharging time (h). As $EV_{l}/M_{l}$ in both V1G and V2G increases, the Natural Gas system capacity significantly reduces at modest EV penetration levels, and remains relatively constant as EV penetration increases. The capacity expansion in VRES + Battery systems significantly reduces at modest EV penetration levels, and gradually continues to decrease as more EVs are added to each system. In V1G, the grid-battery remains present, while V2G rapidly substitutes for the grid-battery. In all cases, cost and total non-curtailed generation decrease monotonically with increased EV penetration, but capacities deployed can increase due to differing capacity factors across technologies.

**Figure 6:**
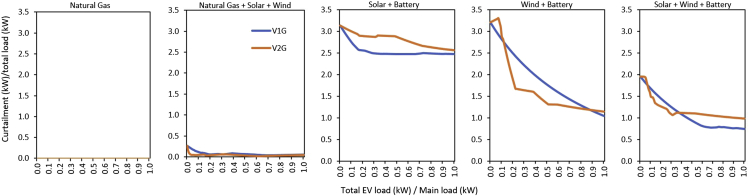
Electricity curtailment: grid curtailment divided by the total load (EV + main) for unidirectional (V1G) and bidirectional (V2G) EV in different electricity systems is shown for the full range of EV-to-main load fractions, which effectively shows the relative amount of wasted generation. There is no curtailed electricity in the gas-only system. In other systems consisting of solar and/or wind, there is curtailed electricity due to the inherent mismatch in VRES generation profiles and electricity demand profiles. Across all VRES + Battery-based systems, curtailment generally declines as EV penetration increases for each system.

Figure 5 shows how the generation capacities of the least-cost electricity systems vary across the full range of $EV_{l}/M_{l}$ considered. In the Natural Gas (dispatchable generator) system (Figure 5), the unused generation capacity was sufficient to cater to the V1G load up to when $EV_{l}/M_{l}$ reaches 0.26. Beyond this point, additional generation capacity is built to cater to the increasing EV load. Relative to V1G, there is a decline in generation capacity as V2G is introduced. However, when $EV_{l}/M_{l}$ reaches 0.26, the generation capacity of V1G and V2G becomes the same up to when $EV_{l}/M_{l}$ reaches 1, as reflected in the electricity cost (Figure 2). One key reason to integrate EVs into electricity systems is their potential ability to utilize excess generation from electricity systems with high amounts of VRES capacity. But for the dispatchable generation system, there was no excess electricity generation (curtailment) (Figure 6). Figure 6 shows the fraction of curtailed generation per unit of supplied load; this is effectively the relative amount of wasted generation and is defined as curtailed generation divided by the total load.

The Natural Gas + Solar + Wind system (Figure 5) exhibits unique attributes. The natural gas generation capacity remains nearly constant across the full range of $EV_{l}/M_{l}$ options for both V1G and V2G, but with lower magnitudes in the entire V2G case, relative to V1G. The model finds it cheaper to invest in natural gas generation capacity, and dispatches it to its emission limits. In V1G, the Natural Gas + Solar + Wind system mainly expands wind while it contracts solar at lower values of$EV_{l}/M_{l}$. As $EV_{l}/M_{l}$ increases, it becomes cheaper to expand solar significantly to provide additional capacity to serve the growing EV stock. When $EV_{l}/M_{l}$ is 1, the wind capacity is more than double the capacity of solar. In the V2G case, attributes similar to the V1G system are observed. These system attributes are further reflected in the electricity curtailment. The stochasticity in both VRES availability, and EV and main load profiles, results in electricity systems with unused electricity generation for portions of the year, which leads to curtailment of generation (Figure 6). The Natural Gas + Solar + Wind system (Figure 6) shows that in the V1G cases, the relative curtailment decreases as $EV_{l}/M_{l}$ increases up to about 0.2, and then begins to increase up to when $EV_{l}/M_{l}$ is about 0.4. This increase in curtailment is mainly driven by the expansion of wind capacity and the limited EV battery capacity. The curtailed electricity in the V2G case shows the same attribute as V1G. However, the curtailed electricity in V2G remains lower for the full range of $EV_{l}/M_{l}$ compared to the V1G case.

The Solar + Battery system (Figure 5) shows that the rate of V1G solar capacity steeply declines to when $EV_{l}/M_{l}$ reaches 0.10, because there is enough curtailed power to satisfy the total load. Beyond this point, the rate of solar expansion is relatively stable for the remaining range of$EV_{l}/M_{l}$. In V2G, the rate of solar expansion remains greater than that of V1G in all cases, because the model builds additional capacity for more curtailed electricity to charge V2G batteries for bidirectional functions. However, as $EV_{l}/M_{l}$ continues to increase, the V2G battery storage is sufficient to provide additional storage space. As EVl/Ml increases, the Solar + Battery system reduces its reliance on expensive grid-battery storage (Figure 5), because of the increasing availability of V2G batteries for the power system. Indeed, we observe that the Solar + Battery system in V2G requires no grid-battery storage when $EV_{l}/M_{l}$ is 0.16 and above (Figure 5). This system attribute is further shown in the grid curtailment (Figure 6), and implies that bidirectional charging EVs do not always reduce VRES capacity, especially in systems consisiting of relatively expensive grid-battery storage.

Figure 5 also shows the capacity expansion in the Wind + Battery system. Here, in the V1G case, curtailed power is enough to serve the full range of loads; thus, the system does not further expand wind capacity, and the rate of storage capacity expansion gently declines for the full range of $EV_{l}/M_{l}$. In the V2G case, the system initially expands wind capacity up to when $EV_{l}/M_{l}$ reaches 0.07. Beyond this point, the rate of wind capacity expansion begins to drop as curtailed power becomes more available to satisfy the loads. The Wind + Battery system in V2G requires no grid-battery storage when $EV_{l}/M_{l}$ is just 0.06 and above (Figure 5). This implies that V2G in the Wind + Battery-dominated system could be more valuable than V2G in the Solar + Battery-dominated system, in terms of grid-battery storage requirements. Figure 6 shows that in V1G cases, curtailment gently decreases as $EV_{l}/M_{l}$ increases to the largest value. However, in the V2G case, curtailed power increases with increasing $EV_{l}/M_{l}$, up to when $EV_{l}/M_{l}$ reaches ∼0.07. Beyond this point, the curtailed power begins to fall, as the system transitions from using electricity from additional capacity installation to using curtailed power.

The Solar + Wind + Battery system displayed different attributes from other systems (Figure 5). In the V1G case, the solar and wind capacity expansion rate declines as $EV_{l}/M_{l}$ increases from 0 to 1, because curtailed electricity is to a large extent sufficient for the loads without a substantial increment in generation capacity (Figures 5 and 6). In the V2G case, the rate of wind capacity expansion gently decreases for the full range of $EV_{l}/M_{l}$, to contract solar capacity as $EV_{l}/M_{l}$ increases. There is no need for grid battery in the Solar + Wind + Battery system when $EV_{l}/M_{l}$ reaches 0.04 (Figure 5).

By the time V2G demand reaches 20% of the main load demand, there is enough EV energy and power capacity to power the system through the night (Figure 5, V2G, Solar + Battery). As V2G capacity increases, there is increasing storage capacity to address wind droughts. Therefore, in the Solar + Wind + Battery case, relative to increasing penetration of V1G, increasing penetration of V2G favors wind over solar because wind is the lowest cost provider of electricity generation.

### Dispatch by EV batteries

**Figure 7:**
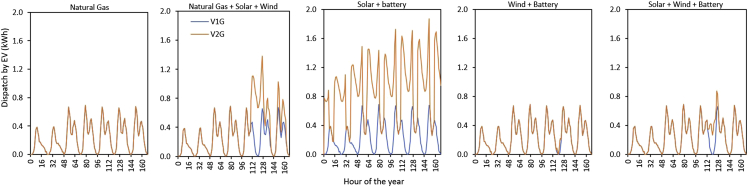
Electricity dispatch by unidirectional (V1G) and bidirectional (V2G) EV in different electricity systems with either V1G or V2G (but not both), when the fraction of EV-to-main load is 0.1. V2G across all electricity systems dispatches more energy compared to V1G, because some energy injects power into the grid at times that allow the system to avoid additional expansion of generation and/or grid-battery capacity.

The EV battery dispatch is obviously limited by transportation demands. Figure 7 shows the EV battery dispatch profile of V1G and V2G for the first week of the year, when the fraction of EV-to-main load ($EV_{l}/M_{l}$) is 0.24. Across all electricity systems considered, the EV battery dispatch by V2G was greater than V1G because of the additional ability of V2G to inject power into the electricity system. Our analysis indicates that the average number of cycles per year for V2G is 48, 60, 89, 59, and 84 for Natural Gas (dispatchable generation), Natural Gas + Solar + Wind, Solar + Battery, Wind + Battery, and Solar + Wind + Battery systems, respectively. Among the five electricity systems investigated, V2G in the Solar + Battery system is the one with the highest number of EV battery cycles. The V2G batteries are primarily employed to replace expensive grid battery every day to handle day-/night-time variations of solar-based systems. This result suggests that V2G deployed in zero-emission grids that are based on VRES and dominated by solar could experience faster degradation, due to frequent charging/discharging, when compared to those deployed in other electricity systems. This degradation could reduce their technical lifetime (Taljegard et al., 2019). In systems dominated by dispatchable generators, where most of the benefits of V2G will come with <10% of the total V2G fleet, battery degradation would affect the 10% V2G penetration. In the case of 100% V2G, that degradation could be spread over all the vehicles, and reduce individual degradation to 1/10 the rate.

### Implications for regional energy systems

As we note earlier, our analysis is not region-specific, but provides a context to understand the value of EV across different regions with varying electricity supply mixes and vehicle stock. To get further insights into our results, we analyze the implication of EVs for a country with an electricity mix similar to one of the scenarios that we already considered. In this regard, we assume the electricity mix remains as it is today and that EVs replace the entire vehicle stock. We consider Qatar, a proxy for countries with 100% dispatchable generation assets (Qatar has 100% natural gas electricity generation assets (Al-Buenain et al., 2021)). Using 196 Wh/km as the average energy consumption of EVs (Electric Vehicle Database, 2021); 16,229 km as Qatar’s approximate average annual vehicle kilometers traveled (Al-Thani et al., 2020), and 1,655,700 units as Qatar’s vehicle stock (CEIC, 2021); we estimate the annual EV load to be 5.3 TWh. As per the IEA energy balances, the total electricity consumption of Qatar was 47.1 TWh in 2019 (IEA, 2021). Considering these values, an EV-to-main load ($EV_{l}/M_{l}$) fraction of 0.11 (Table 2) approximately represents full replacement of Qatar’s ICEV stock with EVs. This depicts an idealized case of Qatar’s electricity system with complete electrification of transport (i.e., 100% dispatchable generation assets and 100% EV). Our analysis shows that in this hypothetical Qatar energy system, the electricity system cost and generation capacity in V2G is about 3.4% and 9.1% lower than in the V1G case, respectively. Thus, deploying bidirectional charging EVs is valuable for Qatar relative to deploying unidirectional charging EVs. This analysis provides a simple framework that can be used to derive high-level insights about the relative value of bidirectional charging EVs for different regions around the world, with varying electricity mixes and vehicle stock. It is worthy to note that this framework does not remove the need for detailed country/regional-level analysis. For such type of analysis, more country/regional-specific techno-economic data will be needed to describe the EV and power system characteristics in detail.

**Table 2:** Estimation of the fraction of EV-to-main load for different countries at 100% EV penetration

Country	Annual vehicle kilometers (km)	2019 Vehicle stock	Total EV load (TWh) (Authors estimate)	2019 Main load (TWh)	Fraction of EV-to-main load (Authors estimate)
Norway	13,100 (Statistics Norway, 2016)	5,173,520 (CEIC, 2021)	13	127 (IEA, 2021)	0.10
Qatar	16,229 (Al-Thani et al., 2020)	1,655,700 (CEIC, 2021)	5	47 (IEA, 2021)	0.11
India	9,765 (Goel et al., 2015)	75,197,000 (CEIC, 2021)	144	1,349 (IEA, 2021)	0.11
USA	18,454 (FHWA, 2018)	276,491,174 (CEIC, 2021)	1,000	4,187 (IEA, 2021)	0.24
Australia	12,100 (Australian Bureau of Statistics, 2020)	18,924,450 (CEIC, 2021)	45	251 (IEA, 2021)	0.18
Ecuador	20,000 (Corral Naveda and Corral Naveda, 2022)	1,764,132 (CEIC, 2021)	7	26 (IEA, 2021)	0.26
Nigeria	17,000 (Cervigni et al., 2013; Dioha and Kumar, 2020)	6,799,586 (CEIC, 2021)	23	27 (IEA, 2021)	0.85

## Discussion

The resulting system cost and capacity expansion for the least-cost electricity system scenarios considered serve as a base for exploring the research question that motivated this paper. Our electricity cost analysis suggests that bidirectional EVs (V2G) are more valuable for a wide spectrum of electrical systems than are unidirectional EVs (V1G). However, this value decreases as the EV stock—$EV_{l}/M_{l}$—increases. In systems consisting of only dispatchable generators, the electricity cost may not fall substantially with high penetration of V2G, as there is limited curtailed electricity for V2G to use. Moreover, such systems are dispatchable, and easily match peak loads without requiring additional electricity from V2G. In systems that include VRES, the electricity cost declines substantially at all penetration levels of V2G relative to V1G. The reason is that V2G reduces the need to build additional expensive storage capacity to match the load at every given hour.

Overall, our findings are in line with some regional studies (Bellocchi et al., 2019a, 2018; Coignard et al., 2018; Dioha et al., 2022a, 2022b; Forrest et al., 2016; Jenn and Highleyman, 2022; Mangipinto et al., 2022; Szinai et al., 2020; Tarroja and Hittinger, 2021; Wolinetz et al., 2018), suggesting that V2G charging strategy could reduce the electricity system cost, when compared to V1G. However, none of these studies characterized how the cost reduction could vary across a wide range of EV loads for different electricity system configurations. This is where our idealized macro-energy system study becomes pertinent, as it allows the exploration of the dynamics, techno-economics, and implications of EVs for a relatively large scale of electricity systems.

Bidirectional EVs could support electricity systems differently. Our results suggest that the initial introduction of V2G, which can supply battery power to the grid, reduces peak residual demand and thus the need to build additional generation capacity (Figure 5). However, as EV penetration capacity increases, and thus overall electricity demand increases, additional introduction of EVs can require expansion of generation capacity in electricity systems dominated by dispatchable generators, and have little impact on the electricity cost. Therefore, most of the benefits from bidirectional EVs could be realized at relatively modest deployment levels in dispatchable-dominated electricity systems. Broadly, the value of bidirectional EVs in power systems dominated by VRES is that they can help reduce investment in generation capacity (Dioha et al., 2022a, 2022b). In addition, our analysis indicates that bidirectional EVs could stimulate investment in VRES, while contracting the capacity of grid-battery storage (Figure 5). An earlier regional study on this subject matter also suggests that bidirectional EVs can increase the share of VRES resources in Hungary (Taljegard et al., 2019).

While acknowledging the superior value of bidirectional EVs in zero-emission grids dominated by VRES, it is also worth noting that bidirectional EVs may experience relatively higher frequency and extent of charging and discharging. In real life, one of the key issues that EV consumers grapple with in terms of bidirectional EVs is whether discharging their vehicle batteries to the grid will be detrimental to their EV batteries because of the additional cycling that will be introduced. A recent study on this subject matter highlighted that to ignore battery degradation when canvassing for bidirectional EVs is not viable (Uddin et al., 2018). Dubarry et al. (2017) examined the battery (Li-ion) implication of bidirectional EVs by selling as much capacity as possible during 1-h periods when the grid needs it the most. They found that additional usage of the batteries, even at constant power, is detrimental to cell performance, and it could shorten the lifetime of battery packs to less than five years. Our results on EVs’ dispatch (Figure 7) suggest that V2G consumers who operate under the Solar + Battery-dominated system could experience more degradation of their EV batteries than would consumers who operate under other types of electricity systems within a given time horizon. This knowledge is pertinent because EV battery degradation is path-dependent. That is, changes in usage patterns could induce a different degradation rate and, consequently, influence the reliability of EV batteries.

Our analysis was based on only grid-responsive unidirectional and bidirectional charging strategies. Therefore, to realize the full system value of V2G charging strategies, EV consumers must be willing to charge and inject power to the grid in a regulated manner. This is pertinent because the status quo of EV charging is largely uncoordinated, unidirectional, and dependent on the habits of the EV owners. The extent to which EV drivers will participate in grid-responsive charging programs would affect the overall value that EVs will provide to the system. This has been shown in a case study for California, a U.S. state with robust EV and decarbonization targets, where it was found that deploying EVs in a light-duty vehicle fleet based on a V2G strategy could enable an additional 11.1% of system-wide electric load to be satisfied by carbon-neutral resources, without substantial changes in the installed generation capacity (Tarroja and Hittinger, 2021). The challenge going forward is to design policies that can increase the propensity of EV consumers to participate in grid-responsive charging programs, based on their local circumstances.

We note that going forward, there will be multiple and simultaneous transitions occurring in the electricity and transportation sectors across different regions. Using Qatar as an example, we show how our analytical framework could be scaled and used to evaluate the relative value of V2G for the other electricity system configurations (Natural Gas + Solar + Wind, Solar + Battery, Wind + Battery, and Solar + Wind + Battery systems). Care needs to be taken while doing so, as there are disparities between the percentage contribution of renewables to electricity mix across various countries, and due to the relative differences in $EV_{l}/M_{l}$ (Table 2). Some regions have weak grid systems that cannot fully integrate EVs. These differences need to be taken into consideration while scaling our results. Countries that design policies based on their circumstances stand the best opportunity to derive the full benefits of V2G.

Currently, EVs are relatively expensive compared to their ICEV counterparts (Adoba and Dioha, 2021; Dioha and Caldeira, 2022; Muratori et al., 2021). For low-income countries, the deployment of EVs and the potential benefits that V2G may deliver will be impracticable given the state of their grids and household incomes. This scenario presents both challenges and opportunities for them. These countries may establish EV targets that can provide a framework for the design of financial incentives to increase consumer EV adoption by reducing upfront cost of EVs, supporting utility planning, and minimizing the fiscal impacts on governments.

What is the implication of our analysis on a global basis? Clearly, there are substantial differences in the value of bidirectional EVs across different jurisdictions, and this makes it difficult to create a generalized statement about how EVs will impact an energy system. The peculiarities of countries create a situation where EVs may be more beneficial for some countries and less beneficial for others, even when they have the same electricity mix. Instance, assuming all EVs are V2G. In terms of electricity cost, regions with 100% dispatchable generation assets with $EV_{l}/M_{l}$ less than 0.26 will find V2G valuable relative to V1G, while regions with the same electricity generation system but with $EV_{l}/M_{l}$ above 0.26 will find V2G of relatively equal value to V1G (Figure 2). Similarly, regions with Wind + Battery-dominated power systems may find EVs in general more valuable in terms of reducing the cost of delivered electricity than would regions with a dispatchable generators-dominated electricity mix (Figure 2). We have not undertaken a detailed analysis for the different regions of the world, and in reality, it may be impossible to have all EV fleets as either V1G or V2G; but the idealized picture we have painted provides an additional context for discussions about the relative value of bidirectional charging EVs globally.

### Conclusion

This study has explored the relative value of bidirectional charging electric vehicles (EVs) versus unidirectional EVs under varying loads for different electricity system configurations, including dispatchable natural gas, solar, wind, and grid-battery storage technologies. We varied the EV-to-main fraction in the modeled systems to understand the techno-economic opportunities associated with increasing EV stock in different electricity generation configurations. Both unidirectional and bidirectional EVs are particularly helpful in zero-emission grids dominated by wind and solar (variable renewable generators) because the EV batteries can be of great help in balancing temporal mismatches in generation and demand. Bidirectional EVs, because of their 2-way charging potential, allow electricity systems to meet peak loads with less generating capacity and thus reduce the average cost of electricity delivered to end-use demand when compared to unidirectional EVs. Because of their potential to feed electricity back to the grid, they also allow electricity systems to have less dedicated grid storage.

Fundamentally, our study suggests that the value of EVs in electricity systems needs to be treated contextually. Carbon-emission-free systems based on variable sources like wind and solar get a great benefit from both support to meet flexible demand, and potential for capacity reduction through bidirectional EV charging. The EV batteries are valuable because they help to compensate for the high degree of variability in wind and solar resources. EV batteries are beneficial to a much lower extent in systems with substantial fossil fuel, because there is a substantial variable (i.e., fuel) cost associated with electricity from these sources, and the EV batteries need to compensate only for variable demand, which today is much less than the variability in wind or solar generation. Irrespective of the electricity system configuration, once you have enough bidirectional EVs to handle peak loads and/or times of low wind and solar generation, the rest of the EVs can be unidirectional, because their main value is providing a flexible load that can use power that would otherwise be curtailed. While numerical values will change from country-to-country because of differences in cost, resource quality, etc., the qualitative conclusions about the value of EVs remain robust.

### Limitations of the study

It should be stressed that this study is dependent on a series of assumptions. Our idealized model has been applied using the German transportation, electricity, and VRES profiles. Beyond having lossless transmission and distribution, the utilization of German profiles alone makes the analysis more realistic for other countries' with resource availability and electricity and driving demand profiles similar to Germany’s. While the focus is on understanding system behaviors, utilizing other country’s profiles would produce results that are numerically different from those presented here. Also, the time-step in our macro-energy system model is 1-h. While this temporal resolution looks reasonable for our analysis, EV load and main load in the real world can vary within minutes and even seconds.

EVs can come in different forms, such as battery electric vehicles (BEVs), plug-in hybrid electric vehicles (PHEVs), and hybrid electric vehicles (HEVs). The analysis presented here assumes that all EVs are BEVs, and they are charged when it is cheapest to buy electricity from the grid. This behavior may be true in an idealized world where consumers try to minimize costs. In practice, EVs can come in different forms and are not always charged when electricity cost is cheapest as human behavior is unpredictable and convenient charging times differ among consumers. Some EV owners prefer to charge their vehicles at night while they sleep, while some prefer charging during the day while they work. If these factors are considered using a dedicated survey, the results of the least-cost electricity systems presented here would most likely be different.

EVs are assumed to be given freely to the power system. In practice, bidirectional charging is usually driven by government policies and incentives. Consumers are generally reluctant to give their EV power to the grid, unless they are incented with benefits that outweigh their need for using EV battery power for domestic purposes. The value of EV in the electricity system could likely reduce if we consider the cost of these incentives. Additionally, while we have tried to capture possible existing and future electricity system configurations, it is worth noting that power system transition pathways hold uncertainties in countries’ future policies and development. We evaluated these electricity system combinations for a single-year analysis, which may differ from how changes will occur over electricity systems for the many future years that it may take for EVs to completely replace ICEVs in the global vehicle stock.

We also note that we modeled dispatchable generators based on the techno-economic characteristics of combined cycle gas turbines. However, combined cycle gas turbines do not reflect the full range of dispatchable generators available in existing or future energy systems. Other available dispatchable generators include single-cycle gas turbines, coal, geothermal, hydro, biomass, and nuclear. If the techno-economic parameters of other dispatchable generators are used, the numerical variations in the value of V1G and V2G presented here could be different, but won’t have a substantial impact on the system behavior. These considerations raise some limitations in our analysis and thus open the window for further studies. While acknowledging the above limitations, we believe that our study provides a good basis for exploring the value of unidirectional and bidirectional charging EVs across a range of electricity system configurations and EV stock.

## STAR★Methods

### Key resources table

**Table:** 

REAGENT or RESOURCE	SOURCE	IDENTIFIER
**Software and algorithms**		

Macro-scale energy model (MEM)	Ken Caldeira’s research group	https://github.com/carnegie/MEM_public/tree/Dioha_et_al_2022
Gurobi optimizer	Gurobi optimization	https://www.gurobi.com/products/gurobi-optimizer/

**Cost assumptions**		

Wind, solar, and natural gas technologies	U.S. Energy Information Administration (EIA)	https://www.eia.gov/outlooks/archive/aeo20/assumptions/pdf/electricity.pdf
Battery technology	Lazard’s levelized cost of storage report	https://www.lazard.com/media/451087/lazardslevelized-cost-of-storageversion-50-vf.pdf

**Input data**		

Weather data	German EnergyPLAN model	https://heatroadmap.eu/energy-models
Electricity demand data	German EnergyPLAN model	https://heatroadmap.eu/energy-models
Transportation demand data	German EnergyPLAN model	https://heatroadmap.eu/energy-models

### Resource availability

#### Lead contact

Further information and requests for resources should be directed to and will be fulfilled by the Lead Author, Michael O. Dioha (mdioha@carnegiescience.edu).

#### Materials availability

This study did not generate new materials.

### Method details

#### Idealized macro-energy system model

Our modeling approach is idealized, and it is based on a least-cost linear optimizer that minimizes the total system cost based on the user-defined constraints (see below for model formulation and below table for model nomenclature). For all technologies included, the model solves hourly for the dispatch and installed capacities. In our model, an unmet demand/lost load is included and characterized by a variable cost of 10US$/kWh. This has been done to ensure that costly electricity demands that occur only a small portion of time do not significantly influence the optimization results. Thus, the electricity produced from all generators as well as storage technologies must always balance with the total electricity sinks (main load, EV load, lost load, and curtailment).

**Table:** Model nomenclature

Symbol	Unit	Description
g	label only	Generation technology (wind, solar, natural gas)
s	label only	Energy storage technologies (Batt., V1G, V2G)
d	label only	Energy storage discharge destinations (grid, V1G driving, V2G driving)
xchargingy	label only	Energy from “x” used to charge storage “y”
xdischargingtoy	label only	Energy from storage “x” discharged to “y”
t	*h*	Time step, starting from 1 and ending at T
$c_{cap}$	$\left(\$/kW_{e}\right)$for generation,$\left(\$/kWh_{e}\right)$ for storage	(Overnight) capital cost
$c_{fixed\;O\&M}$	$\left(\$/yr.kW_{e}\right)$for generation, $\left(\$/yr.kWh_{e}\right)$for storage	Fixed operating and maintenance (O&M) cost
$c_{fixed}$	$\left(\$/h.kW_{e}\right)$ for generation,$\left(\$/h.kWh_{e}\right)$ for storage	Fixed cost
$c_{var}$	$\left(\$/kWh_{e}\right)$	Variable cost (natural gas with CCS)
f	unitless	Capacity factor (generation technology, f = 1 for all t for natural gas)
h	*h/yr*	Number of hours per year (8,784)
i	unitless	Discount rate
n	*yrs*	Asset lifetime
Δt	*h*	Time step size, i.e., 1 h in the model
C	$kW_{e}$ for generation,$kWh_{e}$ for storage	Capacity
$D_{t}$	$kW_{e}$	Dispatch at time step t from generation or energy storage assets
$M_{t}$	$kW_{e}$	Electricity load at time step t (firm, V1G, V2G)
$u_{t}$	$kW_{e}$	Curtailed power
$S_{t}$	$kWh_{e}$	Energy in storage at end of time step t
γ	1/*yr*	Capital recovery factor
δ	1/*h*	Storage decay rate (energy loss per hour) expressed as fraction of energy in storage
η	unitless	Round-trip efficiency (all energy storage assets)
τ	*h*	Storage charging duration

Our model has perfect foresight of energy demand, energy resources availability, and an efficient market coupled with an ideal transmission & distribution (lossless and free). The main decision variables of the model are generation capacity built by each technology and each hourly technology dispatch, which varies each hour continuously to satisfy the loads. The optimization problem was implemented in Python and computed using the Gurobi Optimizer. A link to full details of the input and output data as well as the model code is available at https://github.com/carnegie/MEM_public/tree/Dioha_et_al_2022.

#### Idealized EV component

There are different approaches to modeling electric vehicles based on the research question, and a detailed review of this subject matter is available in the literature (Mahmud and Town, 2016). For region-specific or case study analyses, several parameters such as the number of parked EVs, their state of charge when plugged in, and the EV battery sizes among others come into play. As outlined in the Introduction, our study is idealized, and our focus is to understand dynamic relationships between parameters in order to generate insights into the value of EVs in the context of different stages of transition toward 100% electric mobility. Consequently, we assumed perfect conditions for EV charging within our model, which contains perfect foresight of future electricity costs, and of wind and solar availability. We considered unidirectional and bidirectional EV charging strategies. The unidirectional and bidirectional charging EVs are modeled as a storage technology that charges from the grid when cost-optimal, to later discharge and satisfy the hourly EV load. The bidirectional charging EV is modeled as a storage technology that draws power from the grid to satisfy the EV load and can also discharge power back to the grid when cost-optimal (Mahmud and Town, 2016). It is worthwhile to clarify that both EV charging strategies considered in this study are idealized versions of grid-responsive behavior, and provide an upper bound on the utility and value of these strategies compared to what could realistically be implemented in a real-world setting (Tarroja and Hittinger, 2021). Main simulations contained either unidirectional EV charging or bidirectional EV charging, but not both, and later simulations looked at combined proportions of both charging methods.

Given our idealized optimization approach, EV charging occurs when the cost of electricity is lowest, and power is available. As earlier noted, in a real-world setting, the ability of EVs to respond to electricity prices depend on their state of charge when plugged in, and their ability to sufficiently charge to meet their travel needs. However, because our analysis is stylized and not for a specific case study, we did not consider some of these other parameters that influence EV charging beyond price. In our model, the EV travel needs were delineated with the German hourly transportation demand distribution, which is required to be satisfied at every hour by the EVs. This does not have a material effect on the analysis as most transportation profiles are similar, with morning and evening rush hours. Dispatchable and non-dispatchable generators satisfy the main and EV loads. However, the electricity supplied to the EV load is limited by the EV battery capacity.

#### Model formulation

The complete model formulation and nomenclature is presented below, and it is a modified version of the models presented in (Dowling et al., 2020; Rinaldi et al., 2021; Ruggles et al., 2021; Tong et al., 2020; Yuan et al., 2020).

A capital recovery factor (Equation 1) is used to calculate the fixed hourly costs for the generation assets (wind, solar, natural gas) and the grid battery storage asset (Equation 2) where *n* is based on the lifetime of the associated asset. There is no cost attributed to the EVs nor their energy storage capacity.

(Equation 1) $$\gamma =\frac{i{\left(1+i\right)}^{n}}{{\left(1+i\right)}^{n}-1}$$

(Equation 2) $$c_{fixed,g,Batt}=\;\frac{\gamma c_{cap,g,Batt}+c_{fixed\;O\&M,g,Batt}}{h}$$

The generation and storage capacities are constrained to be greater than or equal to zero for all technologies (Equation 3).

(Equation 3) $$0\leq C_{g,s}$$

At each hour, *t*, the power dispatch from the generation technologies is constrained between 0 and the asset capacity for natural gas and between 0 and the asset capacity times the hourly capacity factor for wind and solar (Equation 4).

(Equation 4) $$0\leq D_{t,g}\leq C_{g}f_{t,g}$$

The charging rates of the three storage technologies are limited by the storage capacities and the storage charging durations for all hours (Equation 5), while the total discharge rates are similarly constrained (Equation 6). The total discharge rate for each storage technology is defined as the sum of its discharge. By definition, storage assets could not discharge to all three locations (grid, unidirectional EV (V1G), and bidirectional EV (V2G)). Thus, one or two of the discharge options listed in Equation 7 were defined as zero for each storage asset: Battery discharged to the grid only, unidirectional EV discharged to supply EV driving demand only, and bidirectional EV discharged both to the grid and to supply EV driving demand.

(Equation 5) $$0\leq D_{t,grid\;charging\;s}\leq \frac{C_{s}}{\tau_{s}}$$

(Equation 6) $$0\leq D_{t,s\;total\;discharge}\leq \frac{C_{s}}{\tau_{s}}$$

(Equation 7) $$D_{t,s\;total\;discharge}=D_{t,s\;discharge\;to\;grid}\;+D_{t,s\;discharge\;to\;V1G}+\;D_{t,s\;discharge\;to\;V2G}$$

The energy stored in any of the storage assets is less than or equal to that technology’s energy storage capacity (Equation 8) and the total dischargeable energy from each storage asset is limited by its storage decay rate (Equation 9).

(Equation 8) $$0\leq S_{t,s}\leq C_{s}$$

(Equation 9) $$0\leq D_{t,s\;total\;discharge}\leq S_{t,s}\left(1-\delta_{s}\right)$$

Energy balance is maintained for all hours for all components of the system throughout each simulation. For all storage assets, energy balance is enforced according to Equations (10) and (11), which also balance the stored energy at the start and end of each simulation. Grid energy balance is maintained according to Equation (12) which includes all sources that can supply the grid, including generation, discharge from the grid battery, discharge to the system from V2G, and discharge from all energy sinks, including the firm load and charging of all storage assets. Equation (13) describes the energy balance for supplying the EV loads.

(Equation 10) $$S_{1,s}=\left(1-\delta_{s}\right)S_{T,s}\Delta t+\eta_{s}D_{T,grid\;charging\;s}\Delta t-D_{T,s\;total\;discharge}\Delta t$$

(Equation 11) $$S_{t+1,s}=\left(1-\delta_{s}\right)S_{t,s}\Delta t+\eta_{s}D_{t,grid\;charging\;s}\Delta t-D_{t,s\;total\;discharge}\Delta t\;t\in 1,\ldots ,\left(T-1\right)$$

(Equation 12) $$\underset{g}{\sum }D_{t,g}\Delta t+D_{t,Batt\;discharge\;to\;grid}\Delta t+D_{t,V2G\;discharge\;to\;grid}\Delta t=M_{t,grid}\Delta t+\underset{s}{\sum }D_{t,grid\;charging\;s}\Delta t+u_{t}\Delta t$$

(Equation 13) $$D_{t,EV\;discharge\;to\;driving}\Delta t=M_{t,EV}\Delta t\;EV\in \left[V1G,V2G\right]$$

The model minimized the system cost for each simulation (Equation 14) by optimizing the installed generation and grid battery storage asset capacities and hourly dispatch.

#### Minimize (*system cost*)

(Equation 14) $$system\;cost\;=\;\underset{g}{\sum }c_{fixed,g}C_{g}+c_{fixed,Batt}C_{Batt}+\underset{g}{\sum }\left(\frac{\sum_{t}c_{var,g}D_{t,g}}{T}\right)$$

The average cost of electricity, or the Levelized cost of electricity (LCOE), was calculated as the system cost divided by the total load (firm load + EV load) for each simulation (Equation 15).

(Equation 15) $$Levelized\;cost\;of\;electricity\;\left(LCOE\right)=\frac{system\;cost}{\sum_{t}\left(M_{t,grid}+M_{t,V1G}+M_{t,V2G}\right)}$$

The installed EV capacity and driving demand were defined for each simulation. Analysis for the EVs was conducted separately for unidirectional and bidirectional EV (i.e., in each simulation, either all EVs are 1-way EVs, or all are 2-way EVs). Simulations contained either unidirectional EV charging or bidirectional EV charging, but not both.

##### Electricity demand, EV demand, and variable renewable energy data

The electricity demand and renewable energy resource distribution profiles were based on the German energy system data, which has been obtained from the EnergyPLAN model files of the Heat Roadmap Europe 4 project (Heat Roadmap Europe, 2021). These files encompass the German (DE) annual hourly electricity demand profile, the annual hourly transportation energy demand profile, and the annual hourly solar and wind resource distribution, which were delineated by hourly time series of capacity factors. We have used the German electricity and energy resources profile from EnergyPLAN because it is widely accessible to the public and thus, improves the transparency of our analysis.

#### Techno-economic data

We used existing technologies as well as present cost estimates for our analysis as outlined in Table 1. Electricity technologies considered in our model include solar PV (with tracking), onshore wind turbine, combined-cycle gas turbine (multi-shaft), and grid-battery storage. The fixed capital cost for each system technology is defined by the purchase cost, installation cost, and the cost of ancillary components. The fixed capital cost and the annual fixed operation & maintenance (O&M) cost were used to calculate the fixed hourly cost. We also included variable O&M and fuel costs accordingly. For the grid-battery storage, we considered a power-to-energy capacity ratio of 1:4 (Lazard, 2019). Accordingly, we divide by $\sqrt{0.9}$ to change the nameplate energy capacity to applicable energy capacity under the assumption that round trip efficiency is 90% with $\sqrt{0.9}$ losses charging and $\sqrt{0.9}$ losses discharging. We compute the fixed O&M cost using the $/kWh 0.25 annual O&M cost and the annual cost of augmentation of 2.5% of initial fixed capital investment plus warranty of 0.8% of the fixed capital investment. The fixed O&M cost was also converted to a cost per usable energy capacity divided by$\sqrt{0.9}$. A monthly self-discharge rate of 1% is applied as per the prevailing market status of Li-ion batteries.

The average battery capacity of EV and average EV electricity consumption is taken as 59.7 kWh and 196 Wh/km, respectively, per the global market trend at the time of writing (Electric Vehicle Database, 2021). No cost is attached to the EV or its battery as they are assumed to be lossless and given freely to the electricity system. The daily distance covered by EVs is ∼33 km, assuming the distance covered per year is 12,000 km (Bäumer et al., 2018). Using an average EV electricity consumption of 19.6 kWh/100 km (Electric Vehicle Database, 2021), we calculated the hourly EV electricity consumption to be 0.268 kWh. EVs’ fixed hourly battery capacity is calculated as 222.35 kWh by dividing the average EV battery size (59.7 kWh (Electric Vehicle Database, 2021)) by the hourly EV electricity consumption. Fast charging (1 h) is assumed for the EV battery charging time (ChargeHub, 2022).

#### Experiment

In this study, we have considered five experimental scenarios for electricity generation systems to capture a range of current and plausible future electricity systems as given below:

• Natural Gas (dispatchable generator)
• Natural Gas + Solar + Wind
• Solar + Battery
• Wind + Battery
• Solar + Wind + Battery

The first type of system considered is the Natural Gas generator-only system to depict regions dominated by dispatchable generators (e.g., Qatar electricity system (IEA, 2021)). As there are different types of dispatchable generators such as coal and nuclear, we used a combined-cycle gas turbine as a proxy for dispatchable generators. The second electricity system considered is the Natural Gas + Solar + Wind system, which depicts most of the energy systems today currently in transition to 100% renewable energy, where solar and wind installations are being supported by existing dispatchable generators (e.g., USA electricity system). In this case, the least-cost model favors the deployment of the natural gas generator, and consequently, an emission reduction constraint was imposed on the natural gas generator to allow the implementation of solar and wind.

The third system considered is the Solar + Battery system to depict future power systems near-fully, or fully, powered by solar PV and grid-battery storage, especially in regions with good solar resources. This type of system can also depict current solar PV systems dedicated to EV charging. The fourth system configuration is the Wind + Battery system to depict future power systems near-fully, or fully, powered by wind and grid-battery storage, especially in regions with good wind resources. The fifth type of system considered is Solar + Wind + Battery to elucidate future power systems near-fully, or fully, run on solar and wind energy tied to a grid battery to provide the needed grid balance.

These experiments are not intended to predict future energy systems for different countries, as there are profound differences among the operations of the different dispatchable and variable renewable energy generators, and the levels of EV integration, in different regions. A divergent and more important purpose of these experiments is to understand how the value of bidirectional charging EVs could vary under the unique features of different electricity system configurations relative to the value of unidirectional EVs.

As earlier noted, the stock of EV could impact the value of EV in electricity systems as there may be substantial differences in EV load across different regions on account of regional differences in vehicle ownership rate. An increase in EV deployment will lead to an increase in existing electricity demand. In one high-end scenario, widespread EV and other demand-side electrical technologies could lead to a 40% increase in the current US electricity demand by 2050 (Mai et al., 2018). It is pertinent to consider scenarios of different EV loads and how they could impact different electricity systems. In this context, for each electricity system configuration modeled, we considered different cases for the fractions of EV-to-main load ($EV_{l}/M_{l}$) at 100% EV deployment level. This refers to the total EV electricity demand divided by the total electricity demand of the main load (Equation 16).

(Equation 16) $$\text{EV}-\text{to}-\text{main load fraction}=\frac{EV\;electricity\;demand}{Main\;load}$$

As per Equation (16), we estimated a hypothetical $EV_{l}/M_{l}$ (assuming all ICEVs today are replaced with EVs) for different regions using 196 Wh/km as the average energy consumption of EVs (Electric Vehicle Database, 2021), average annual vehicle kilometers traveled (km), vehicle stock (CEIC, 2021), and the total electricity consumption (TWh) of each region (IEA, 2021) as presented in Table 2.

From Table 2, the $EV_{l}/M_{l}$ value of 0.11 approximates Qatar at 100% electrification based on their current vehicle ownership rate, while it corresponds to the United States at around 50% conversion of its ICEVs to EVs. Consequently, this type of approximation can be used to get high-level estimates of $EV_{l}/M_{l}$ for different regions at different stage of EV adoption. The $EV_{l}/M_{l}$ analyzed ranged from 0 (i.e., only main load is available) to 1 (i.e., 100% EV load is equal to the main load). The analysis was done in fraction steps of 0.01, resulting in 101 cases (Ruggles et al., 2021). For all cases, the main and EV load profiles were kept constant.

## Data Availability

• Input data (wind and solar resources, electricity demand, and transportation demand) required to reproduce the results reported in this paper were retrieved from the German EnergyPLAN model files of the Heat Roadmap Europe 4 project (Heat Roadmap Europe, 2021). • Code required to reproduce the results reported in this paper are available in the GitHub repositories at https://github.com/carnegie/MEM_public/tree/Dioha_et_al_2022. • Any additional information required to reanalyze the data reported in this paper is available from the lead contact upon request.
